# A novel method of measuring passive quasi-stiffness in the first metatarsophalangeal joint

**DOI:** 10.1186/s13047-016-0173-2

**Published:** 2016-10-26

**Authors:** Marabelle L. Heng, Yaohui K. Chua, Hong K. Pek, Priathashini Krishnasamy, Pui W. Kong

**Affiliations:** 1 Physical Education and Sports Science Academic Group, National Institute of Education, Nanyang Technological University, 1 Nanyang Walk, Singapore, 637616 Singapore; 2 Podiatry Department, Singapore General Hospital, Diabetes & Metabolism Centre, 17 Third Hospital Avenue, Singapore, 168752 Singapore; 3 Sports Medicine and Surgery Clinic, Tan Tock Seng Hospital, Medical Centre 2, 11 Jalan Tan Tock Seng, Singapore, 308433 Singapore

**Keywords:** Force, Torque, Video, Angular displacement, Mobility, Tactile pressure

## Abstract

**Background:**

First metatarsophalangeal joint (MTPJ) mobility is commonly assessed by its angular displacement (joint angle) or subjectively rated as ‘hypermobile’, ‘normal’ or ‘stiff’ by a clinician. Neither of these methods is ideal because displacement alone does not take into account the force required to displace the joint and subjective evaluation is not always reliable. This study presented a novel method to determine the passive quasi-stiffness of the first MTPJ. The reliability of the proposed method was also assessed. The first MTPJ passive quasi-stiffness of 13 healthy subjects were measured at two occasions, 7 days apart, by two testers (experienced and inexperienced). A tactile pressure sensing system was used to measure the force applied to dorsiflex the first toe by the testers. The torque (in Nmm) about the first MTPJ was calculated as the applied force (in N) multiplied by a moment arm (in mm), where moment arm was the length of the first proximal phalanx. A video camera recorded the motion of the first MTPJ, simultaneously with force measurements, to determine the joint angular displacement (in degrees) using the Dartfish software. The quasi-stiffness (in Nmm/degrees) was calculated as the slope of a graph where torque was plotted against first MTPJ angular displacement. Descriptive statistics of the first MTPJ quasi-stiffness were calculated. Intra-rater and inter-rater reliability were assessed using Bland and Altman plot, intraclass correlation coefficients (ICC), and standard error of measurement (SEM).

**Results:**

First MTPJ quasi-stiffness of the subjects ranged widely from 0.66 to 53.4 Nmm/degrees. Intra-rater reliability for experienced tester was moderate (Session 1: 14.9 ± 14.6 Nmm/degrees, Session 2: 14.2 ± 8.5 Nmm/degrees, ICC = .568, SEM = 7.71 Nmm/degrees). Inter-rater reliability between experienced (12.6 ± 8.4 Nmm/degrees) and non-experienced (19.9 ± 9.2 Nmm/degrees) testers was poor (ICC = -.447, SEM = 11.29 Nmm/degrees).

**Conclusions:**

First MTPJ passive quasi-stiffness can be quantified from torque and angular displacement measurements using simple equipment in a clinical setting. The tester’s experience affected the consistency in joint quasi-stiffness measurements.

**Electronic supplementary material:**

The online version of this article (doi:10.1186/s13047-016-0173-2) contains supplementary material, which is available to authorized users.

## Background

The first metatarsophalangeal joint (MTPJ) is the articulating joint between the first metatarsal and the proximal phalanx of the big toe. It is classified as a single synovial joint that allows motion in the sagittal and transverse planes, with sagittal plane dorsiflexion being the joint’s primary movement in gait. Approximately 65 to 75 degrees of dorsiflexion is considered necessary to enable efficient forward transfer of bodyweight during the propulsive phase of gait [1, 2]. Restricted first MTPJ range of motion can alter foot function, leading to first MTPJ pain and the development of secondary conditions such as plantar calluses and inefficient gait [1–4]. On the opposite end of the mobility spectrum, a hypermobile or unstable first MTPJ may be associated with conditions such as hallux valgus (a forefoot deformity commonly known as “bunion”) and metatarsalgia (pain under the ball of foot) [5–7]. Clinically, first MTPJ mobility is assessed in cases where there are foot propulsion problems (e.g., hallux limitus). Other uses of measuring MTPJ range of motion include screening and identification of people who may be at high risk of diabetic foot ulceration [8] and assessment of surgery outcome in patients treated for hallux valgus [9].

There are no standardised methods for measuring first MTPJ mobility. A common practice is to subjectively “feel” how easily the first MTPJ moves through its range of motion [10, 11]. The end-of-range quality is often described using various adjectives such as “bony block” or “normal” by a clinician. Hypermobile joints have been documented in literature as a joint with “excessive excursion with soft end-point” [12]. This subjective assessment method is descriptive and not quantifiable. First MTPJ range of motion is also sometimes visually estimated by a clinician or measured using goniometric devices. Unfortunately, neither visual estimation nor goniometric measurements are sufficiently reliable to be deemed clinically acceptable regardless of the experience of the testers [13]. Research studies have reported wide variations in the range of motion for the first MTPJ—dorsiflexion: 65 to 110 degrees; plantarflexion: 23 to 45 degrees [2, 14–16]. One reason for the discrepancy in the range of motion is the different “zero” or starting positions for measurement of joint motion. A second factor is that the first metatarsal shaft naturally plantarflexes when the hallux dorsiflexes, shifting the reference point and hence causing large variations in the measurements. Thirdly, the amount of force applied to displace a joint to maximum range is tester-dependent and thus range of motion is not entirely objective [17]. It is possible that one tester uses slightly more force than another tester, resulting in greater range of motion.

Quasi-stiffness, which is the resistance of a joint to an external force, may be a better alternative to assess the first MTPJ mobility. Quasi-stiffness is often determined experimentally as the derivative of the torque-angle relationship [18, 19]. Although quasi-stiffness and stiffness are conceptually different by definition [18], they are mechanically equivalent when the system is behaving with passive dynamics [19]. Previous studies have reported methods in measuring the passive stiffness of other foot joints including the first ray [12, 20, 21] and the ankle [22]. There are, however, no published procedures for the measurement of the first MTPJ quasi-stiffness.

Quantifying first MTPJ quasi-stiffness can be useful in clinical assessment as well as informing orthotic prescription. With quasi-stiffness measurements, joint mobility can be assessed in an objective manner instead of relying on subjective descriptions such as “excessive excursion with soft end-point” [12]. For example, when evaluating a patient with hallux valgus, it is recommended that the first MTPJ be assessed for quantity and quality of motion [23]. Quasi-stiffness measurement of the first MTPJ can accurately reflect how stiff or hypermobile the joint is. Currently, there are methods to assess the extent of hallux valgus deformity (e.g. Manchester scale [24]) but the biomechanical risk factors associated with the development of hallux valgus is not well studied [25]. Quantifying the quasi-stiffness of the first MTPJ may provide useful information on how susceptible a joint is to developing hallux valgus, and hence contributing to screening and early intervention. In the treatment of hallux rigidus and hypermobile first MTPJ, a morton’s extension splint of varying stiffness (rigid or semi-rigid) is the acceptable orthotic modification to ‘splint’ the first MTPJ so as to restrict motion. However, the decision as to whether or not to restrict motion and by how much (choice of rigid or semi rigid) depends largely on the clinician’s experience and subjective assessment of the situation. In orthotics prescription, the clinician also makes a decision on orthotic shell stiffness (e.g. very stiff carbon fiber for more control or thin polypropylene for less stiffness and control). Such prescription is often based on trial-and-error, subjected to the clinician’s assessment. It is common that orthotics prescribed require further adjustments, which may signify that initial assessments or prescriptions may not have been accurate [26, 27]. Therefore, the underpinning motivation of this study was to develop a method for quantifying first MTPJ quasi-stiffness which may, in a long term, lead to better treatment and more accurate prescription of orthotic devices.

This study aimed to present a novel method incorporating joint range of motion and force applied to displace the first MTPJ, in order to measure passive quasi-stiffness of the first MTPJ. The intra-rater and inter-rater reliability of the proposed method were also assessed.

## Methods

Thirteen healthy participants [7 men, 6 women, mean (*SD*) age = 24.4 (5.0) years, body mass = 63.3 (15.1) kg, height = 1.67 (0.11) m] were recruited for this study. All participants had no joint conditions (such as inflammatory arthritis and osteoarthritis), had no previous foot surgeries or trauma, and were free from foot pain and injuries at the time of study. They were informed of the nature, benefits, and risks of the study, and provided their written consent for participation. Ethical approval was received from the Nanyang Technological University Institutional Review Board (IRB/2013/08/18).

Participants were assessed for the first MTPJ quasi-stiffness in their dominant foot on two occasions (Sessions 1 and 2), 7 days apart. In Session 1, MTPJ quasi-stiffness was assessed by an experienced podiatrist (MH) who has a Bachelor of Podiatry (Australia) and 6.5 years of clinical experience. In Session 2, the measurements were taken by the same experienced podiatrist as well as an inexperienced tester (YC) who had no prior knowledge of podiatry and had only underwent two training sessions pertaining to measurement procedures used in this study. The measurement orders of experienced and inexperienced testers were randomised.

**Fig. 1:**
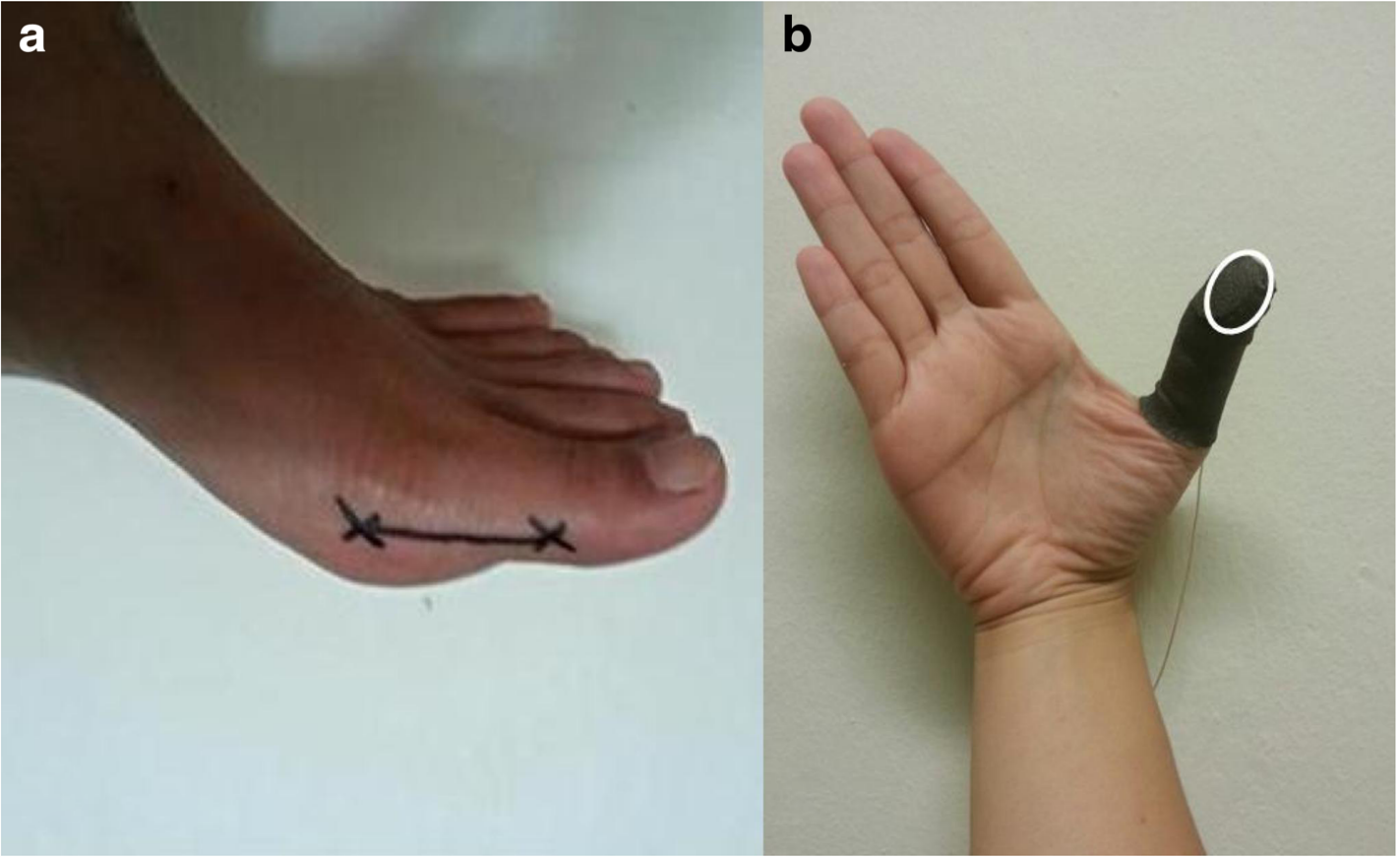
**a** Moment arm for first metatarsal, represented by line drawn from first metatarsal head tuberosity to beneath tuberosity of distal phalanx. **b** FingerTPS sleeve with sensor pad (*circled in white*) over thumb

**Fig. 2:**
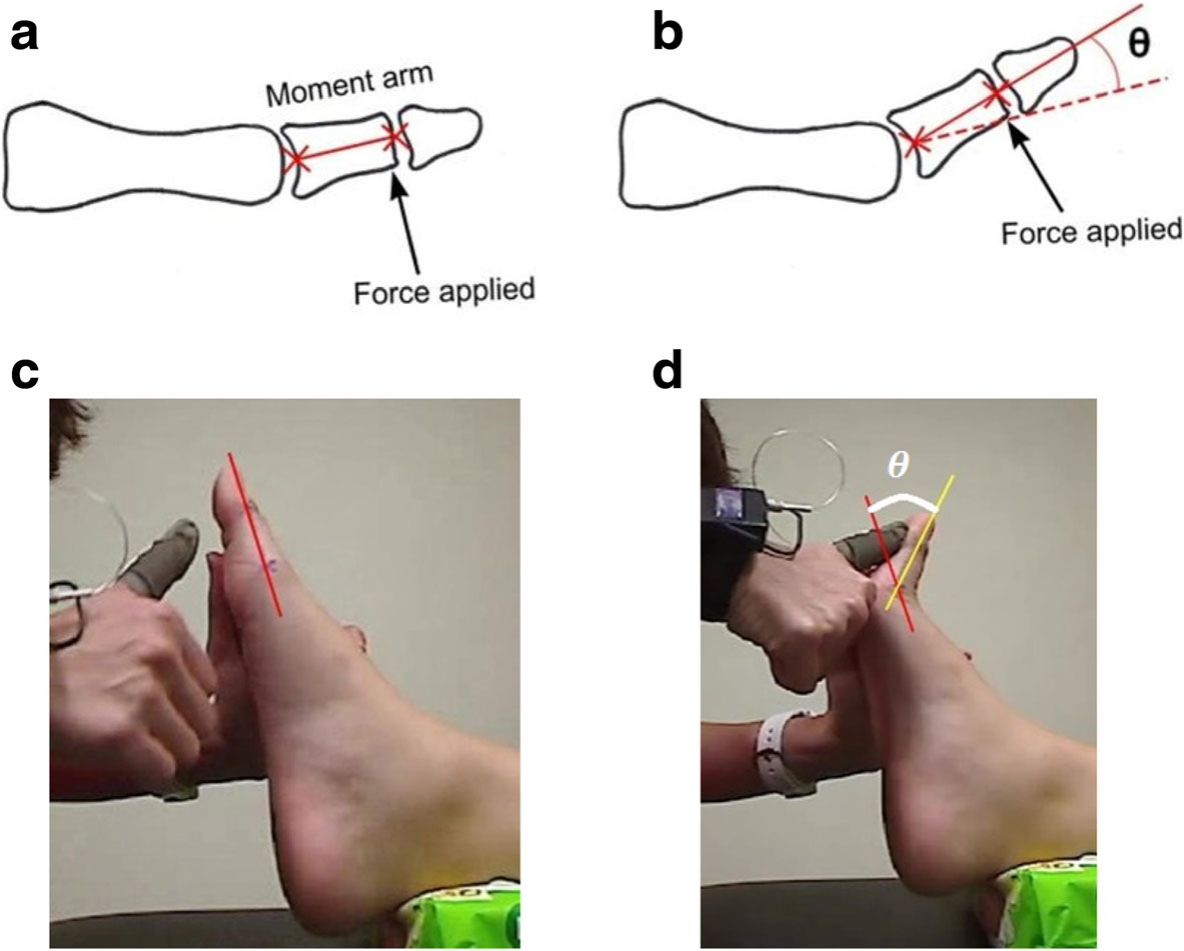
**a** Moment arm for first MTPJ is length of proximal phalanx. **b** Perpendicular force applied to the proximal phalanx dorsiflexes the first MTPJ. θ is angular displacement in degrees. The dotted line represents the resting position of the proximal phalanx or the “zero” reference point. **c** Still image from video recording capturing showing line of resting position. **d** Still image from video recording showing angular displacement, θ

First MTPJ mobility was assessed with the participants in a non-weight-bearing position. Participants were asked to lie on the examination table in supine position, with a soft block placed just proximal to the Achilles area of the dominant leg. The foot was held at the ankle in its neutral positon by the tester’s non-working hand. The full range of motion of the first MTPJ was initially identified by the tester by moving the hallux into maximal dorsiflexion and back to the neutral starting positions. A line representing the moment arm was marked from the tuberosity of the first metatarsal head to just beneath the tuberosity of first distal phalange in the medial aspect of the foot (Fig. 1a). The moment arm was measured in millimetres (mm). To minimise parallax error, a 2 MP (1600 × 1200 pixels) resolution camera (Logitech Web Pro 9000, Logitech, CA, USA; Carl Zeiss Lens Tessar 2.0/3.7) was set up perpendicular to the side of the foot. Hence it can be assumed that the dorsiflexion movement of the first MTPJ occurs in a plane perpendicular to the optical axis of the camera. Measurement of force applied to move the first toe into dorsiflexion was done using the Finger TPS tactile pressure sensing system (Pressure Profile System Inc., Los Angeles, CA, US). With Finger TPS pressure sensor fitted on the thumb of the tester’s working hand (Fig. 1b), a perpendicular force was applied in the direction of dorsiflexion, under the “x” marking of the distal end of the first proximal phalanx, producing rotation about the first MTPJ (Fig. 2). The camera recorded the angular displacement of the first MTPJ in synchronization with the Finger TPS system at 25 Hz. Dartfish video analysis software (Dartfish Ltd., Fribourg, Switzerland) was later used to determine the joint angular displacement (joint angle). In order to check the accuracy of the angle measurements, a goniometer displaying a series of angles (5°, 15°, 25°, 35°, 45°, 55°, order randomised) was videotaped. These angles were manually digitised twice in Dartfish by the inexperienced tester (YC) who was blinded from the goniometric readings. The root mean squared (RMS) difference was 2 degrees across all angles when compared to actual goniometric angles.

**Fig. 3:**
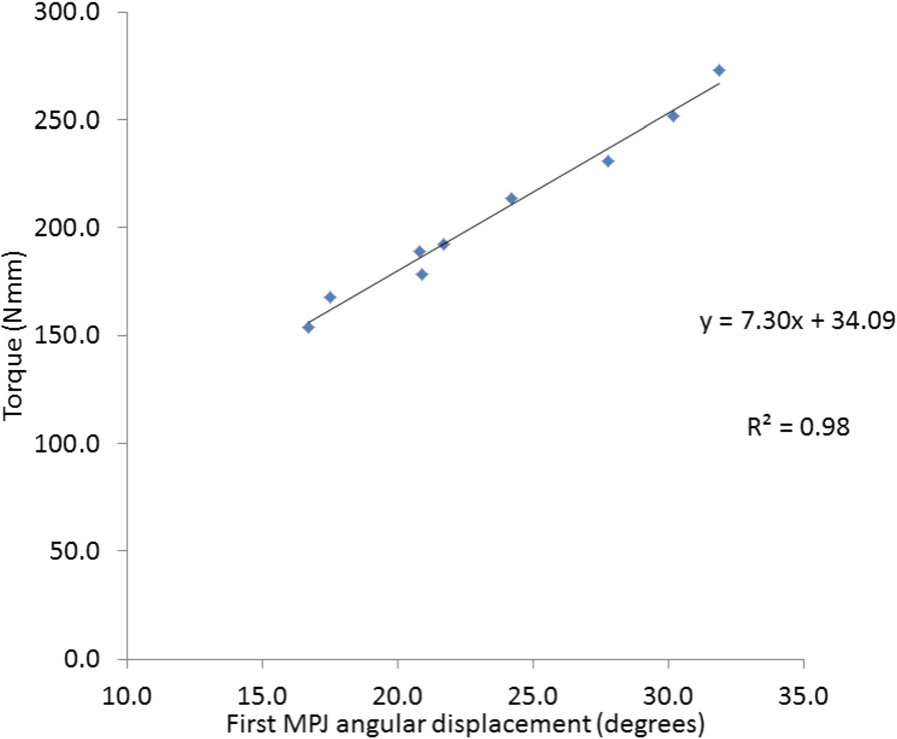
Plot of torque (in Nmm) against first MTPJ angular displacement (in degrees). The slope of the graph (7.30 Nmm/degrees) is the quasi-stiffness of the joint. The R^2^ indicates that 98 % variability can be explained by this model

The process of measuring first MTPJ range of motion was as follows: a) The first MTPJ was moved into maximal dorsiflexion passively by the tester; this data point was excluded in the analysis because joint quasi-stiffness close to the end range cannot be assumed linear [22]; b) The force applied to the first toe was slowly released, allowing the toe to move from maximum dorsiflexion back towards the starting neutral position; c) The tester paused briefly for about 2 seconds at three intermediate positions to allow static measurement of torque and angular displacement [(T_1_,θ_1_), (T_2_,θ_2_), (T_3_,θ_3_)] before returning the big toe to its neutral position; and d). This process was repeated three times providing a total of nine data points [(T_1_,θ_1_), … (T_9_,θ_9_)] to plot a torque-angular displacement graph (Fig. 3).

The quasi-stiffness (k) of the first MTPJ joint can be calculated based on the following equation:

$$ \mathrm{T}\kern0.5em  = \mathrm{k}\ \uptheta $$

where T = Torque = Force (in N) x Moment Arm (in mm), and θ = angular displacement (in degrees). By plotting a torque-angular displacement graph, the MTPJ quasi-stiffness was determined as the slope of the plot (Fig. 3).

Statistical analysis was performed using IBM SPSS Statistics 21 (Armonk, NY) and Microsoft Excel. The first MTPJ quasi-stiffness was the only dependent variable. Descriptive statistics were used to summarise the group data in means and standard deviations. Repeated measurements which were performed twice by the experienced tester were used to assess between-day intra-rater reliability using the Bland and Altman plot and intra-class correlation coefficient [ICC (3,1)]. The ICCs were interpreted as slight (<.20), fair (.21–.40), moderate (.41–.60), substantial (.61–.80), and almost perfect (> .80) [28]. The standard error of measurement (SEM) was also calculated to provide an estimate of the amount of error associated with the measurement. Similarly, inter-rater reliability between the experienced and inexperienced testers was assessed using Bland Altman plots, ICC and SEM. Due to technical error in video recording, we were unable to determine the angular displacement data of three participants assessed by the inexperienced tester. Hence, inter-rater reliability analyses were performed using ten participants’ data.

## Results

**Fig. 4:**
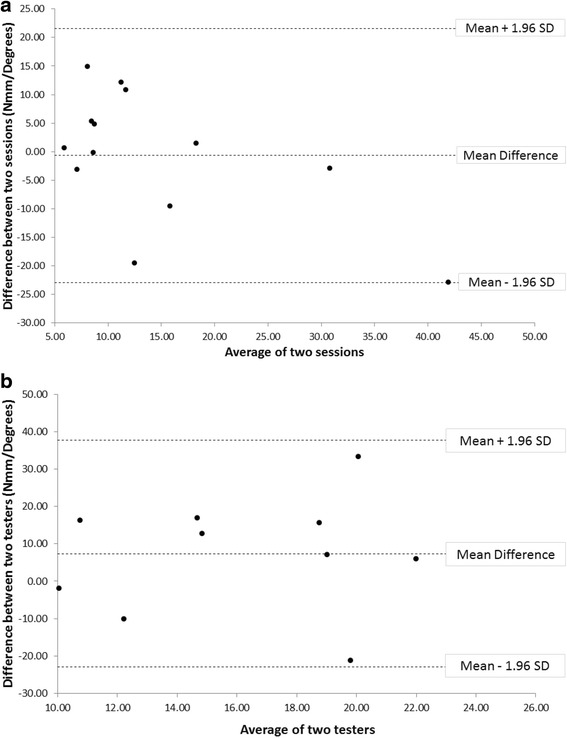
**a** Bland-Altman plot for measurement of the first metatarsophalangeal joint (MTPJ) quasi-stiffness assessed on two different sessions by the same experienced tester, and **b** the same session by two different testers (experienced versus inexperienced)

First MTPJ dorsiflexion angles (θ_1_, …,θ_9_) used to plot the torque-angular displacement graphs ranged between 3.2 to 47 degrees (median = 24 degrees). Table 1 illustrates the descriptive and reliability statistics results. Overall, there was a large variation in the measured MTPJ quasi-stiffness values, ranging from 0.66 to 53.4 Nmm/degrees. The between-day intra-rater reliability for the experienced tester was moderate (ICC = .568). Although the mean difference between Session 1 and Session 2 was small as a group (0.68 Nmm/degrees), the measurement error was rather large with a SEM of 7.7 Nmm/degrees. Visual inspection of the Bland Altman plot showed random scatter of the points between the limits of agreement, indicating homoscedastic data as well as an absence of systematic bias (Fig. 4a).

**Table 1:** Intra-rater (*n* = 13) and inter-rater (*n* = 10) reliability of first metatarsophalangeal joint (MTPJ) quasi-stiffness

Rater	Mean ± *SD* (Range) [Nmm/degrees]	Mean ± *SD* (Range) [Nmm/degrees]	Mean Difference ± *SD* [Nmm/degrees]	SEM [Nmm/ degrees]	ICC
Intra-rater	Session 1	Session 2			
	14.9 ± 14.6	14.2 ± 8.5	-0.68 ± 11.4	7.71	.568
	(0.66–53.4)	(2.72–30.5)			
Inter-rater	Experienced	Inexperienced			
	12.6 ± 8.4	19.9 ± 9.2	7.36 ± 15.7	11.29	-.447
	(2.72–30.5)	(7.14–36.7)			

Mean Difference was calculated from Mean (Session 2)—Mean (Session 1) or Mean (Inexperienced)—Mean (Experienced)

*SD* standard deviation, *SEM* standard error of measurement, *ICC* intraclass correlation coefficient

When comparing the MTPJ quasi-stiffness measurements between the experienced and inexperienced testers, the inter-rater reliability was poor (Table 1). The poor agreement between the two raters was reflected by the ICC (-.447), large mean difference (7.36 Nmm/ degrees) and large measurement error (SEM = 11.3 Nmm/degrees). Although ICC is commonly interpreted between 0 and 1, ICC as a correlation can be negative when the variance within subjects is greater than that for the raters [29]. Visual inspection for the Bland Altman plot showed random scatter of the points between the limits of agreement, indicating homoscedastic data as well as an absence of systematic bias (Fig. 4b).

## Discussion

This study reported a novel method to quantify first MTPJ quasi-stiffness in a clinical setting using pressure sensors and video analysis. The between-day intra-rater reliability was moderate for the experienced tester but the inter-rater reliability was poor between the experienced and inexperienced testers.

### Quantifying joint quasi-stiffness

Given that our study is the first to measure first MTPJ passive quasi-stiffness, there are no available data for direct comparison in the literature. Thus, the study findings will be discussed in reference to relevant studies on the stiffness of other foot joints including the first ray [20] and ankle [22, 30].

In the study by Glasoe et al [20], the first ray (metatarso-cuneiform) stiffness was calculated from the slope of the force-linear displacement graph. Their study found an association between increased first ray stiffness and positive prayer sign which is an indication of contracture and stiffness of finger joints [31, 32]. Glasoe et al [20] reported the first ray stiffness (measured at 2 mm linear displacement) for diabetes participants with and without positive prayer sign as 19.0 (9.6) N/mm and 15.9 (7.9) N/mm, respectively. Our present study adopts a similar approach in assessing joint quasi-stiffness by taking into consideration both the force applied to move the joint and resulting displacement, calculating the quasi-stiffness from the slope of a torque-angular displacement graph. This approach which quantifies quasi-stiffness of a foot joint has also been applied at the ankle [22, 30]. Trevino et al [22] reported ankle stiffness of healthy subjects as 60 ± 40 Nmm/degree; this is about 4 times the mean first MTPJ quasi-stiffness in our participants (≅14.6 Nmm/degree) measured by the experienced tester. This difference appears reasonable considering that the ankle is a larger and more complex joint with more ligaments, tendons, and larger muscles around the joint as compared to the first MTPJ. Major source of passive tension is thought to be brought about by myofibrillar structure proportional to muscular size, therefore muscle size is positively correlated with passive quasi-stiffness [30].

In studies examining passive ankle stiffness, the torque versus angular displacement plots were non-linear throughout the measured range [22, 30]. As the range of motion approaches the end range, the stiffness becomes non-linear and increases (steeper gradient). In recognition of the varying stiffness throughout the range of motion, Salsich et al [30] calculated two stiffness values for the ankle joint separately for the first and second halves of the passive torque curve. In the present study, we measured the MTPJ passive linear quasi-stiffness near the mid-range portion, avoiding the maximum end range of motion. This approach is similar to the ankle stiffness calculated over the ‘working range-of-motion’ in the study by Trevino et al [22].

The novel method presented in this study was developed for use in a clinical setting. The Finger TPS system and camera were portable and easy to be placed next to an examination table. This set-up does not interfere with the space required in normal clinical assessments: the camera is small and mounted on a tripod, while the pressure sensor is worn on the tester’s thumb and linked to the laptop via wireless receiver. The time required for data collection from each participant was about 10 minutes, inclusive of preparation and explanation. Analysis was performed offline after the participant has left. Digitisation of video recordings to determine angular displacement and extraction of corresponding force data from the software took about 10 minutes per participant. All equipment and software used are commercially available, with a total cost of approximately USD 7500.

### Reliability

The reliability of measuring first MTPJ passive quasi-stiffness using the methods described in this study appears unsatisfactory. Measurement errors were, at best, moderate even for the experienced tester, with a large SEM of 7.71 Nmm/degree (Table 1). The low reliability may be attributed to the following potential variations: (i) The force data obtained using the tactile pressure sensing system may not be sufficiently accurate. While we calibrated the sensor following standard procedures for each test session, the force measured is dependent on the location of pressure pad over thumb at the point of force application. During measurement, pressure pad location may have shifted and the force applied may not be directly perpendicular to the movement lever arm, which may interfere with assumptions of the joint movement mechanics; (ii) There may be errors associated with the camera position and manual digitisation to determine angular displacement. To minimise parallax error, we carefully placed the camera perpendicular to the side of the foot to allow a sagittal view of the MTPJ dorsiflexion. The small error in manual digitisation (RMS difference = 2 degrees) also reassures the quality of the angular displacement data obtained. (iii) The foot was held in neutral position by the non-working hand instead of being secured in a foot clamp. This may contribute to variations with subtle shifts in foot positions when measurements were taken; (iv) First metatarsal movement was not considered in this study due to the simplified protocol; this movement may result in variation in first MTPJ dorsiflexion angles; (v) The data range may have exceeded the ‘working range’ where quasi-stiffness can be assumed linear [22]. As the joint moves closer to the end range, the quasi-stiffness (gradient of graph) increases with the range of motion.

When similar methods were carried out by the inexperienced tester, the measurement error was even greater (SEM = 11.29 Nmm/degree). This is likely due to the lower proficiency in securing the foot joints and manipulating the toe movement. Comparing the outcomes between the inexperienced tester to experienced tester, we speculated that the differences were observed because the experienced tester is able to better control the amount of force applied to the joint. This allows the experienced tester to manipulate the joint movements within the ‘working range’ where the quasi-stiffness is assumed to be linear and less variable. The inexperienced tester, who may have difficulty in controlling such fine movements, may have over-displaced the joint near the end range where quasi-stiffness becomes non-linear and highly variable [22]. Overall, our results suggest that clinical experience plays a role in subjective assessments of first MTPJ quasi-stiffness.

### Future directions

To overcome the issues associated with clinical experience and human error, it will be necessary to develop a device that can quantify first MTPJ quasi-stiffness. Future directions for development can consider the following stages: (i) Technology for accurate quantification of foot joint quasi-stiffness in a clinical setting, (ii) commercialisation of equipment, (iii) gathering normative data to determine cut-off values for diagnoses for hallux conditions associated with joint quasi-stiffness, and (iv) targeted, precision treatment based on objective measurements.

Current subjective evaluation methods used by clinicians are unreliable [13]. In its place, there should be a clinical device to objectively quantify joint quasi-stiffness. Previous studies have measured joint stiffness for the first ray, forefoot and ankle joints in research environments [20, 22, 30], but the practice of objective measurements of quasi-stiffness has not been implemented in clinical settings. An affordable, easy-to-use device to measure small joints quasi-stiffness is needed.

With future population level studies and normative data, classification of pathological foot conditions is recommended. There is potential for advancing care to enable early intervention in conditions associated with abnormal joint quasi-stiffness. Currently, objective measurement as a method of assessing possible early pathological first MTPJ conditions does not exist. For instance, hallux valgus (bunions) is diagnosed after joint deformation has occurred, described as “medial slant of first metatarsal” [33].

Interventions for joint hypermobility can be more specific and targeted. Currently, the orthotic stiffness required to control for hypermobile joints in the foot is estimated by clinicians based on their experience. With objective measurements, deviations from normal joint quasi-stiffness may be corrected with corresponding material stiffness.

## Conclusions

This study provided preliminary results that first MTPJ passive quasi-stiffness can be quantified from torque and angular displacement measurements using simple equipment in clinical settings. The tester’s experience affects the consistency in joint quasi-stiffness measurements. Future studies can aim at refining the measurement protocol, and to develop instrumentation that can accurately quantify foot joint quasi-stiffness.

## Additional file

Additional file 1: MTPJ quasi-stiffness supporting data. (XLSX 14 kb)

## Abbreviations

ICC: Intraclass correlation coefficient
MTPJ: Metatarsophalangeal joint
SEM: Standard error of measurement
